# Preoperative vitamin D insufficiency increases the risk of delayed neurocognitive recovery via acute systemic inflammation in elderly women undergoing gynecological surgery

**DOI:** 10.3389/fmed.2026.1626647

**Published:** 2026-02-18

**Authors:** Ju Bao, Di Zhu, Hua-Ping Zhao, Ye Lu, Dong-Liang Mu, Ting Ding

**Affiliations:** 1 Department of Anesthesiology, Peking University First Hospital, Beijing, China; 2 Department of Anesthesiology, The First Affiliated Hospital of Zhengzhou University, Zhengzhou, Henan, China; 3 Department of Obstetrics and Gynecology and Reproductive Medicine, Peking University First Hospital, Beijing, China; 4 Outcomes Research Consortium, Cleveland, OH, United States

**Keywords:** acute systemic inflammation, delayed neurocognitive recovery, elderly woman, mediation analysis, vitamin D insufficiency

## Abstract

**Background:**

Delayed neurocognitive recovery (DNR) is common among elderly women following gynecological surgery. This patient group is also prone to vitamin D insufficiency (VDI). Present study was designed to investigate the association between preoperative VDI and DNR in elderly female patients undergoing major gynecological surgery.

**Methods:**

In this prospective cohort study, elderly women (≥65 years) scheduled for major gynecological surgery under general anesthesia were enrolled. The primary outcome was the incidence of DNR on the fifth day, which was assessed using the Montreal cognitive assessment (MoCA). VDI was defined as serum 25-hydroxyvitamin D levels <50 nmol/L. The acute change in systemic inflammatory response was reflected by the ratio of postoperative neutrophil-to-lymphocyte ratio (NLR) to preoperative NLR. The association between VDI and DNR was analyzed using multivariable logistic regression. Mediation analysis was conducted to explore the relationships among VDI, acute systemic inflammation, and DNR.

**Results:**

A total of 156 patients were enrolled with mean vitamin D concentration 37.6 ± 18.7 nmol/L. 115 (73.7%) patients were classified as VDI. VDI patients suffered higher incidence of DNR than non-VDI patients [22.6% (26/115) vs. 7.3% (3/41), *p* = 0.035]. After adjustment of confounders, both VDI (OR 4.905, 95% CI 1.079–22.307, *p* = 0.040) and postoperative NLR/preoperative NLR (OR 3.775, 95% CI 1.398–10.192, *p* = 0.009) which reflect acute systemic inflammation change were associated with an increased risk of DNR. Mediation analysis showed that the effect of VDI on DNR was significantly mediated by acute systemic inflammation (adjusted β 3.2, 95% CI 0.000 to 7.000%, *p* = 0.045) which accounted for 15.2% of the total effect.

**Conclusion:**

Among elderly women, preoperative VDI correlates with an elevated risk of DNR after major gynecological surgery. Acute systemic inflammatory responses may have served as a partial mediator in this correlation.

**Clinical trial registration:**

www.chictr.org.cn, identifier ChiCTR2000033130.

## Introduction

Early postoperative neurocognitive disorders include DNR and postoperative delirium (POD) (1). DNR is common in patients after gynecological surgery; it primarily manifests as an impairment of cognitive and memory function, execution disability, and attention deficit (2–4). In elderly women, its incidence may reach up to 40% (5). DNR is associated with poor patient outcomes including an increase in mortality and morbidity, a decline in daily activity, and an increase in healthcare costs (6–8).

Vitamin D is a fat-soluble vitamin that plays an important role in the nervous system. Serum 25-hydroxyvitamin D (25-OHD) is the standard clinical marker used to assess a person’s overall vitamin D status. About approximately 75% of postmenopausal women are affected by VDI (9). In elderly individuals, the incidence of VDI is as high as 90% (10). The association between a lower vitamin D level (25-OHD < 32.4 nmol/L) and an increased risk of cognitive impairment has been established in population-based studies (11). A systematic review demonstrated that vitamin D deficiency (25–50 nmol/L) was associated with 34% elevated risk with cognitive impairment (12). Recent cohort studies of patients undergoing major cancer surgery have reported that elderly patients with preoperative VDI were at higher risk of DNR (13, 14).

Although elderly women are prone to VDI and constitute a high-risk group for DNR, the association between vitamin D and cognitive function in this group is uncertain. Several studies have reported that VDI was associated with an increased risk of cognitive impairment in elderly women (15–17). However, one Canadian cohort reported that a higher 25(OH)D concentration (i.e., 69.4 nmol/L vs. 23.4 nmol/L) is associated with an increased risk of dementia in women but not in men (18). The association between VDI and DNR in women undergoing surgery also lacks evidence.

In recent decades, an increasing body of research has demonstrated that Vitamin D exerts broad anti-inflammatory and immuno-modulatory effects on various human extra-skeletal systems (19, 20). VDI may exacerbate inflammageing and non-specific inflammation observed in older adults (21, 22). VDI may also activate chronic cerebral inflammatory response which is considered as the main risk factor of dementia (23). Meanwhile, acute systematic inflammation may exacerbate neuro-inflammation and enhance cognitive impairment (24). Surgery-induced inflammation, the underlying mechanism of DNR, is manifested by acute elevation of systematic markers such as NLR (25). However, it is unclear if acute systemic inflammation plays a role in the association between VDI and DNR.

The present study was designed to investigate whether preoperative VDI is associated with DNR in elderly women undergoing gynecological surgery.

## Methods

### Study design and ethics

This prospective cohort study was conducted in Peking University First Hospital (a tertiary general hospital). The study protocol was approved by Biomedical Ethics Committee of Peking University First Hospital (No. 2019-331, April 24, 2020, Chairman: Xiao-hui Guo), and was registered with Chinese Clinical Trial Registry on May 21, 2020 (www.chictr.org.cn; ChiCTR2000033130). Written informed consent was obtained from all participants. First patient was enrolled on June 9, 2020.

### Participants recruitment

Elderly women (age ≥65 years old) who were scheduled for major gynecological surgery under general anesthesia were enrolled.

Patients were excluded if they met with any of the following criteria: (1) expected surgery duration less than 1 h or hysteroscopy; (2) vitamin D supplementation within recent 3 month; (3) neuropsychological diseases such as dementia and schizophrenia; (4) American Society of Anesthesiologist physical status classification IV or above; (5) refused to take part in this study.

### Vitamin D measurement

Patients generally needed to be admitted to the hospital 2–5 days prior to surgery, depending on their comorbidities. With an overnight fasting, a venous blood sample was collected from each participant at the day before surgery, late in the morning. Liaison XL 25(OH)D assay (DiaSorin, Stillwater, United States) was used to measure serum 25(OH)D concentration with the analytical range between 10 nmol/L and 375 nmol/L and variation coefficient between 5.04 and 6.14%. External quality assurance was 100% which was acceptable for the quality control.

According to global consensus, VDI was diagnosed if 25(OH)D ≤ 50 nmol/L (26).

### Neutrophil/lymphocyte ratio

Neutrophil/lymphocyte ratio was tested at the morning on preoperative 2–3 days and postoperative first day, respectively.

### Perioperative management

Eligible patients were visited at 1 day before surgery. After obtaining written informed consent, baseline characteristics were collected, including demographic data, comorbidities, serum 25(OH)D concentration, NLR, and MoCA.

All patients received standard monitoring including heart rate and rhythm, blood pressure, peripheral arterial oxygenation, Bispectral Index (BIS), end-tidal carbon dioxide, nasopharyngeal temperature, and urine output. Propofol (2–4 mg/kg for induction followed by 4–10 mg/kg/h for maintenance) and remifentanil (targeted concentration effect 2–4 ng/mL) were used for general anesthesia. Sevoflurane and sufentanil were used if necessary. Muscle relaxation was maintained via the intermittent injection of rocuronium. The aim of anesthesia was to keep systolic blood pressure at ≥80% the baseline reference, a BIS of 40–60, and nasopharyngeal temperature 36–37 °C.

Patient-controlled intravenous analgesia (PCIA) was provided. Sufentanil 1 μg/h was continuously infused with an additional bolus of 2 μg and a block time of 8 min. Pain intensity was assessed using an 11-score numeric rating scale (NRS, 0 indicating no pain and 10 indicating the worst pain). The aim of PCIA was to keep the NRS ≤ 3. Flurbiprofen 50 mg per se or parecoxib 40 mg per se were used as a supplementation to the analgesia.

### Outcomes

#### Primary outcome

The primary outcome was the incidence of DNR 5 days postoperatively. To assure data quality, MoCA was administrated by a designated anesthesiologist (Dr. Ju Bao) for cognitive function evaluation at 1 day before surgery and at postoperative fifth day, respectively. All patients completed cognitive assessment and subsequent follow-up. MoCA is a well-validated screening instrument designed to detect individual at risk for mild cognitive impairment (MCI) (27). The full version of MoCA was specifically developed as a point-of-care assessment that can be administrated in approximately 10 mins by a trained healthcare professional after completing a 1 hour training module (27). Given its ease of use and sensitivity, MoCA was selected as a feasible tool for use in a busy surgical ward setting in current research. DNR was established if postoperative MoCA was 2 scores lower than the preoperative baseline (28, 29).

#### Secondary outcomes

Postoperative delirium (POD) was evaluated twice daily using the confusion assessment method (CAM) for non-intubated patients and CAM-intensive care unit (CAM-ICU) for intubated patients from the first to the fifth day postoperatively (30). The research department possesses considerable expertise in delirium assessment and has established a robust training system for assessors through its prior research endeavors (31, 32). Investigator who performed postoperative follow-up were trained by a psychiatrist to use the CAM and CAM-ICU to assess delirium. The initial training required that delirium diagnosis has 100% agreement between investigators and psychiatrist with simulated patients.

Non-delirium postoperative complications within 30 days postoperatively that needed medical treatment (i.e., Clavien-Dindo classification of surgical complications grade II or above) were collected (33). We also collected the length of the postoperative in-hospital stay and medical expense incurred during hospitalization.

### Statistical analysis

#### Sample size calculation

The incidence of VDI in surgical women was about 70% and the incidence of DNR was about 30% in these patients, whereas the incidence of DNR was about 10% in those without VDI Taking significance level at 0.05 and power at 0.8, 152 patients were needed with the participants ratio of 3:1 in VDI group and control group. Present study planned to enroll 160 patients with considering a follow-drop rate of 5%.

### Data analysis

The normality of the continuous data was tested using a histogram. Normal data were presented as the mean ± standard deviation (SD) and were compared using an independent sample *t*-test; otherwise, they were presented as the median (interquartile range, IQR) and compared using an independent sample Mann–Whitney test. Categorical data were presented as the number (percentage) and were compared using Chi-square or Fisher’s exact tests.

Patients were divided into a VDI group and a control group according to their preoperative serum vitamin D concentration. Concerning the primary outcome, the incidence of DNR between the two groups was compared using a Chi-square test; a relative risk with 95% confidence interval (CI) was calculated. Categorical secondary outcomes were analyzed with the same method including the incidences of POD and non-delirium complications. The length of postoperative in-hospital stay and medical expenses were analyzed using a Mann–Whitney test; the differences between two groups were estimated using the Hosmer–Lemeshow test.

A univariate analysis was used to screen for potential risk factors of DNR. Variables with *p* ≤ 0.1 and factors with clinical significance (i.e., age, ASA, and surgery time) were entered into a multivariable logistic regression analysis to identify the independent risk factors for DNR.

The severity of changes in acute systemic inflammation was defined as the ratio of postoperative NLR/preoperative NLR. Patients were divided into two groups according to whether or not the severity of change was higher than 1.86 (the cutoff value for the risk of developing DNR in patients without VDI derived from Receiver Operating Characteristic analysis).

Mediation analysis was employed to investigate whether the causal effect of VDI on DNR (path d) could be apportioned into an indirect effect (path a*b, mediated by acute systemic inflammation) and a direct effect (path c) (34). We defined an indirect effect as the change in the expected incidence of DNR when the status of VDI was fixed, but the status of acute systemic inflammation changed as if the status of VDI was changed. We defined direct effect as the change in expected incidence of DNR when the status of VDI changed, but the status of acute systemic inflammation was artificially fixed. We modelled acute systemic inflammation using a logistic regression, with status of VDI as an independent variable. We modelled the incidence of DNR using a logistic regression, with status of VDI and acute systemic inflammation as independent variables. The total, indirect, and direct effects were quantified using the “mediation” package (35). The coefficients of indirect effect estimated the strength of the mediated effect of VDI on DNR, that is, how much of the increase in DNR that occurs as VDI was due to acute systemic inflammation. The coefficient of direct effect estimated the strength of the direct effect of VDI on DNR, that was, any effect of VDI on DNR that was not mediated by acute systemic inflammation. The coefficient of total effect was the sum of the direct and the mediated effect. Bootstrapping (5,000 iterations) was used to estimate CIs. Missing data was not imputed, and the number of missing cases was reported in tables with brackets.

## Results

### Patient population and baseline variables

**Figure 1:**
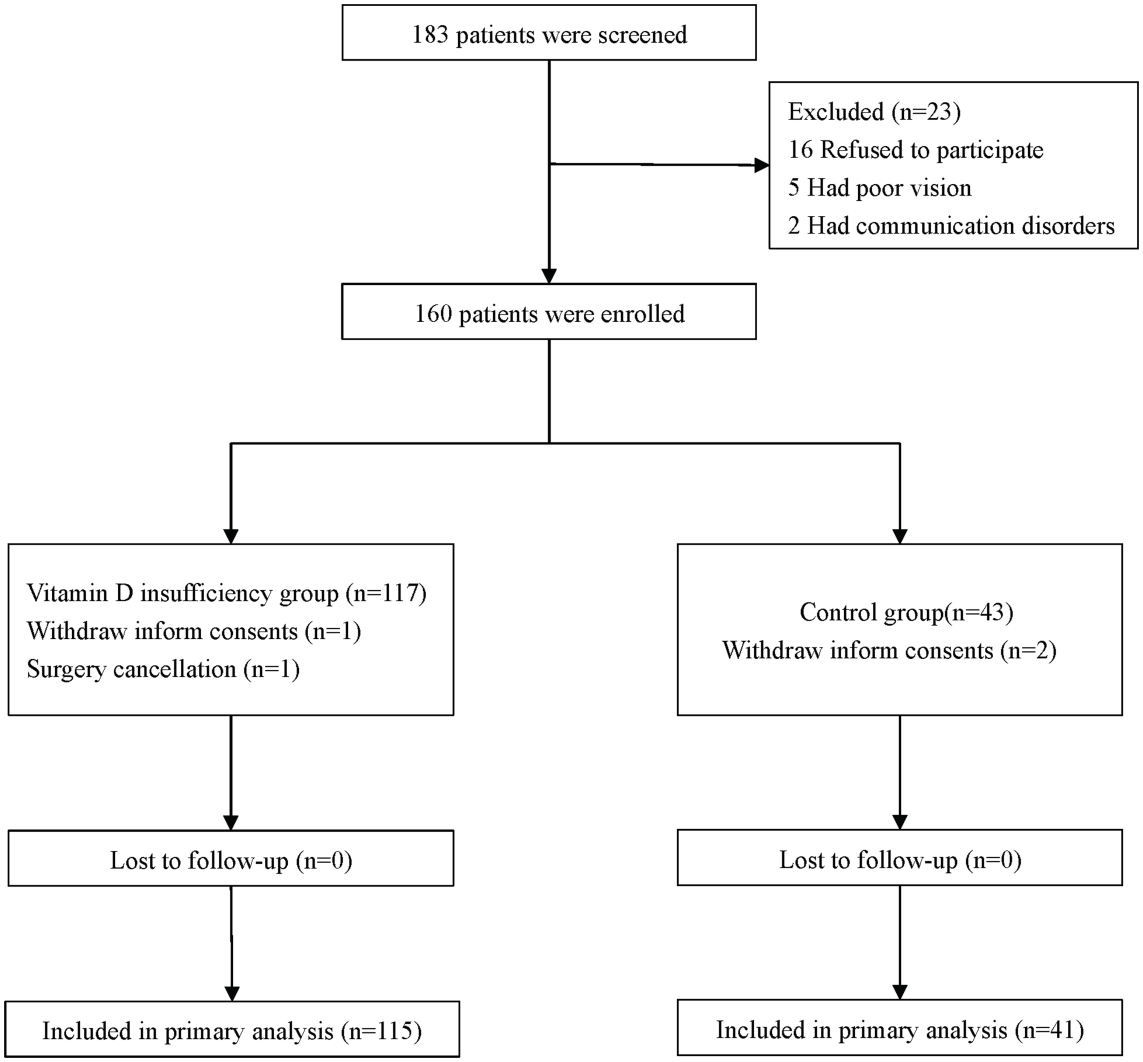
Flowchart.

From 9 June 2020 to 3 January 2022, 183 patients were screened; of these, 160 patients were enrolled (Figure 1). Four patients were excluded because of withdrawn informed consent or surgery cancellation. All patients completed cognitive assessment and follow-up. No patient died within 30 postoperative days.

The mean age of the enrolled patients was 70.5 ± 4.7 years, with a mean vitamin D level of 37.6 ± 18.7 nmol/L (Table 1). In total, 73.7% (115/156) of them were classified as VDI, with a mean vitamin D level of 28.7 ± 9.0 nmol/L. The mean vitamin D level in the control group was approximately 62.8 ± 15.9 nmol/L.

**Table 1:** Baseline variables.

Variable	All patients (*n* = 156)	VDI group (*n* = 115)	Control group (*n* = 41)	*p*-value
Age (year)	70.5 ± 4.7	70.7 ± 4.9	70.0 ± 4.0	0.462
Body mass index (kg/m^2^)	24.8 ± 3.3	25.1 ± 3.4	23.9 ± 3.1	0.050
Education level				**0.992**
Illiterate	12 (7.7%)	8 (7.0%)	4 (9.8%)	
Elementary school	14 (9.0%)	11 (9.6%)	3 (7.3%)	
Middle school	48 (30.8%)	35 (30.4%)	13 (31.7%)	
High school	44 (28.2%)	34 (29.6%)	10 (24.4%)	
College and above	38 (24.4%)	27 (23.5%)	11 (26.8%)	
ASA classification				**0.508**
II	96 (61.5%)	69 (60%)	46 (40%)	
III	60 (38.5%)	27 (65.9%)	14 (34.1%)	
Marriage				**0.169**
Married	137 (87.8%)	104 (90.4%)	33 (80.5%)	
Divorced	4 (2.6%)	3 (2.6%)	1 (2.4%)	
Widowed	15 (9.6%)	8 (7.0%)	7 (17.1%)	
Preoperative comorbidities				
Stroke	14 (9%)	12 (10.4%)	2 (4.9%)	0.358
Anxiety^*^	2 (1.3%)	1 (0.9%)	1 (2.4%)	0.458
Depression^*^	3 (1.9%)	2 (1.7%)	1 (2.4%)	1.000
Coronary artery disease	22 (14.1%)	17 (14.8%)	5 (12.2%)	0.683
Hypertension	88 (56.4%)	68 (59.1%)	20 (48.8%)	0.251
Arrhythmia^†^	16 (10.3%)	12 (10.4%)	4 (9.8%)	1.000
Asthma	1 (0.6%)	1 (0.9%)	0 (0%)	1.000
Diabetes	39 (25%)	29 (25.2%)	10 (24.4%)	0.916
Thyroid disease	8 (5.1%)	7 (6.1%)	1 (2.4%)	0.682
Hyperthyroidism	1 (0.6%)	1 (0.9%)	0 (0.0%)	0.549
Hypothyroidism	7 (4.5%)	6 (5.2%)	1 (2.4%)	0.461
Hyperlipidemia	38 (24.4%)	28 (24.3%)	10 (24.4%)	0.996
Musculoskeletal disorders^‡^	22 (14.1%)	13 (11.3%)	9 (22.0%)	0.093
Barthel index, score^§^	98.6 ± 4.6	98.3 ± 5.2	99.3 ± 2.4	0.277
Duration of daily activity				**0.104**
<0.5 h	83 (53.2%)	66 (57.4%)	17 (41.5%)	
0.5–2 h	62 (39.7%)	40 (34.8%)	22 (53.7%)	
≥2 h	11 (7.1%)	9 (7.8%)	2 (4.9%)	
Calcium supplementation (%)^‖^	30 (19.2%)	16 (13.9%)	14 (34.1%)	0.005
MoCA, score^#^	22 ± 4	22 ± 4	22 ± 3	0.898
MoCA score < 26	116 (74.4%)	84 (73.0%)	32 (78.0%)	0.529
Preoperative neutrophil/lymphocyte (NLR)^**^	2.2 (1.7, 3.0)	2.3 (1.8, 3.0)	2.0 (1.5, 2.8)	0.162
Vitamin D (nmol/L)	37.6 ± 18.7	28.7 ± 9.0	62.8 ± 15.9	< 0.001
Calcium (mmol/L)	2.4 ± 0.1	2.4 ± 0.1	2.3 ± 0.1	0.356
Magnesium (mmol/L)	0.9 ± 0.1	0.9 ± 0.1	0.9 ± 0.1	0.267
Phosphorus (mmol/L)	1.1 ± 0.2	1.2 ± 0.2	1.1 ± 0.2	0.288
Hemoglobin (g/L)	128.5 ± 12.4	127.9 ± 13.3	130.2 ± 9.4	0.326

Data were presented as mean ± SD, *n* (%) or median (interquartile range). ASA, American Society of Anesthesiologist; MoCA, Montreal cognitive assessment.

^*^Diagnosed by psychiatrists.

^†^Including premature ventricular complexes, premature atrial contraction, and I^o^ atrioventricular block.

^‡^Including spinal spondylolysis and joint osteoarthrosis.

^§^A 100-score scale with higher score for better daily activity.

^‖^Defined as regular intake of calcium supplementations within recent 3 months.

^#^A 30-score scale with higher score for better cognitive function.

^**^The NLR is a reproducible marker of systemic inflammatory responses.

The baseline variables were comparable between the VDI group and the control group, except for the lower daily intake of calcium in VDI patients [13.9% (16/115) vs. 34.1% (14/41), *p* = 0.005].

The proportion of cancer surgery was higher in patients with VDI than in the control group [41.2% (47/115) vs. 19.5% (8/41), *p* = 0.013] (Table 2). The proportion of patients with a postoperative NLR/preoperative NLR ≥ 1.86 was higher in the VDI group than in the control group [58.3% (67/115) vs. 36.6% (15/41), *p* = 0.017]. VDI patients suffered from a slightly higher pain intensity during movement within the first 3 days postoperatively compared to the control group (all *p*-values <0.05). Other perioperative variables were similar between the two groups, including the type of anesthesia, the duration of surgery, and intraoperative adverse events (Table 2).

**Table 2:** Perioperative variables.

Variable	All patients (*n* = 156)	VDI group (*n* = 115)	Control group (*n* = 41)	*p*-value
Anesthesia type				0.782
General anesthesia (GA)	146 (93.6%)	108 (93.7%)	38 (92.7%)	
GA + paravertebral block	10 (6.4%)	7 (6.1%)	3 (7.3%)	
Duration of anesthesia (min)	195 ± 66	197 ± 67	188 ± 61	0.452
Duration of surgery (min)	141 ± 57	144 ± 61	133 ± 46	0.151
Type of surgery				0.585
Uterus	23 (14.7%)	15 (13.0%)	8 (19.5%)	
Ovarium	19 (12.2%)	16 (13.9%)	3 (7.3%)	
Uterus combined Ovarium	99 (63.5%)	73 (63.5%)	26 (63.4%)	
Other gynecological surgery	15 (9.6%)	11 (9.6%)	4 (9.8%)	
Cancer surgery	55 (35.3%)	47 (41.2%)	8 (19.5%)	0.013
Intraoperative medications				
Propofol (mg)	560 (413, 700)	550 (430, 713)	570 (386, 674)	0.316
Sufentanil (μg)	20 (15, 25)	20 (15, 25)	20 (15, 25)	0.120
Remifentanil (μg)	698 (507, 1,040)	700 (530, 1,040)	624 (447, 1,043)	0.629
Use of etomidate	150 (96.2%)	111 (96.5%)	39 (95.1%)	0.654
Use of midazolam	147 (94.2%)	109 (94.8%)	38 (92.7%)	0.621
Use of sevoflurane	111 (71.2%)	86 (74.8%)	25 (61%)	0.094
Total fluid input (mL)	1,000 (1,000, 2000)	1,500 (1,000, 1,500)	1,000 (1,000, 1,500)	0.221
Estimated blood loss (mL)	50 (50, 100)	50 (30, 100)	50 (50, 100)	0.781
Urine output (mL)	350 (200, 550)	350 (200, 500)	400 (200, 650)	0.195
Intraoperative adverse events				
Hypotension	105 (67.3%)	77 (66.9%)	28 (68.3%)	0.876
Hypoxemia	3 (1.9%)	3 (2.6%)	0 (0.0%)	0.567
Hypertension	29 (18.6%)	23 (20.0%)	6 (14.6%)	0.448
Postoperative analgesia^*^				0.401
None	14 (9%)	9 (7.8%)	5 (12.2%)	
PCIA	142 (91.0%)	106 (92.2%)	36 (87.8%)	
Consumption of sufentanil (μg)	29.0 (24.5, 36.0)	29.0 (24.5, 35.5)	29.0 (25.0, 36.0)	0.721
Use of NSAIDs	47 (33.3%)	31 (29.5%)	16 (44.4%)	0.101
ICU admission	1 (0.6%)	1 (0.6%)	0 (0.0%)	0.549
Pain intensity at movement^†^				
Postoperative first day	4.0 (2.0, 5.0)	4.0 (3.0, 6.0)	3.0 (1.5, 5.0)	0.013
Postoperative Second day	3.0 (1.0, 4.0)	3.0 (2.0, 5.0)	2.0 (1.0, 3.0)	0.008
Postoperative third day	1.0 (0.0, 3.0)	2.0 (0.0, 3.0)	1.0 (0.0, 2.0)	0.040
Sleep quality^‡^				
Postoperative first night	5.7 ± 2.5	5.9 ± 2.5	5.3 ± 2.4	0.233
Postoperative second night	7.1 ± 1.7	7.0 ± 1.8	7.3 ± 1.7	0.338
Postoperative third night	7.9 ± 1.7	7.8 ± 1.7	8.1 ± 1.5	0.485
Postoperative neutrophil/lymphocyte (NLR)^§^	4.2 (2.9, 6.5)	4.3 (3.3, 6.8)	4.1 (2.5–6.4)	0.214
Postoperative NLR/preoperative NLR^‖^ ≥1.86	82 (52.6%)	67 (58.3%)	15 (36.6%)	0.017
MoCA, score^#^	22.7 ± 4.0	22.7 ± 4.2	22.7 ± 3.4	0.996

Data were presented as mean ± SD, *n* (%) or median (interquartile range). PCIA, Patient-controlled intravenous analgesia; NSAIDs, Non-steroid anti-inflammatory drugs; ICU, Intensive care unit.

^*^PCIA was provided at the discrete of patients. For patients who refused to use PCIA, intravenous or oral NSAIDs were provided in necessary.

^†^Pain intensity was assessed by numeric rating score (11-score, 0 for no pain and 10 for worst pain).

^‡^Sleep was assessed by numeric rating score (11-score, 0 for worst sleep and 10 for good sleep).

^§^The NLR is a reproducible marker of systemic inflammatory responses.

^‖^Acute systemic inflammation was defined as the ratio of postoperative NLR/preoperative NLR ≥ 1.86 (the cutoff value for the risk of developing DNR in patients without VDI derived from Receiver operating characteristic analysis).

^#^A 30-score scale with higher score for better cognitive function.

### Primary outcome

The overall incidence of DNR was 18.6% (29/156). Patients with VDI suffered from a higher incidence of DNR than those in the control group [22.6% (26/115) vs. 7.3% (3/41), RR = 3.700, 95% CI 1.056–12.967, *p* = 0.035] (Table 3).

**Table 3:** Primary and secondary outcomes.

Variables	VDI group (*n* = 115)	Control group (*n* = 41)	*p*-value
Primary outcome			
DNR	26 (22.6%)	3 (7.3%)	0.035
Secondary outcomes			
POD	3 (2.6%)	0 (0.0%)	0.567
Non-delirium complications^*^	32 (27.8%)	11 (26.8%)	0.746
Postoperative nausea and vomiting	23 (20%)	9 (22%)	0.823
Respiratory complications^†^	2 (1.7%)	0 (0.0%)	>0.99
Anastomotic leakage	1(0.9%)	0 (0.0%)	>0.99
Unplanned reoperation^‡^	4 (3.5%)	0 (0.0%)	0.574
Systemic inflammatory response syndrome	2 (4.9%)	2 (1.7%)	0.275
Length of postoperative in-hospital stay (day)	12 (7, 14)	13 (11, 13)	0.905
Medical expense (thousand yuan)	30 (23, 38)	26 (23, 30)	0.137
Death within postoperative 30 days	0 (0.0%)	0 (0.0%)	NA

Data were presented as mean ± SD, *n* (%) or median (interquartile range). DNR, Delayed neurocognitive recovery; POD, Postoperative delirium; NA, Not available.

^*^Including postoperative nausea and vomiting, respiratory complications, anastomotic leakage, unplanned reoperation, and systemic inflammatory response syndrome.

^†^Including atelectasis and acute respiratory failure.

^‡^Including major bleeding and incisional hernia.

### Secondary outcomes

The incidence of POD was similar between the two groups [2.6% (3/115) vs. 0.0% (0/41), *p* = 0.567] (Table 3). The incidence of non-delirium complications was comparable between the two groups (*p* = 0.746). The length of postoperative in-hospital stay and medical expenses incurred were also comparable between the two groups [12 (7, 14) days vs. 13 (11, 13) days, *p* = 0.905] and [30 (23, 38) thousand-yuan vs. 26 (23, 30) thousand-yuan, *p* = 0.137].

### Association among VDI, postoperative NLR/preoperative NLR ratio, and DNR

In the univariate analysis, four variables with *p* ≤ 0.1 were identified as potential risk factors of DNR (Supplementary Table 1). After an adjustment of the above confounders and predefined factors, a multivariable logistic analysis showed that VDI was associated with an increased risk of DNR (OR = 4.905, 95% CI 1.079–22.307, *p* = 0.040) (Table 4). The ratio of postoperative NLR/preoperative NLR ≥ 1.86 was also associated with an increased risk of DNR (OR = 3.775, 95% CI 1.398–10.192, *p* = 0.009).

**Table 4:** Risk factors of DNR by multivariable logistic regression analysis.

Variable	Univariate analysis		Multivariable analysis	
	Odds ratio (95% CI)	*p*-value	Odds ratio (95% CI)	*p*-value
Age (per year increase)	1.007 (0.925–1.096)	0.873	—	—
Education level (per level increase)^*^	0.759 (0.542–1.063)	0.109	—	—
ASA physical status classification (per level increase)	1.162 (0.511–2.642)	0.721	—	—
Preoperative MoCA (per score increase)^†^	0.891 (0.805–0.987)	0.027	0.895 (0.805–0.994)	0.038
Duration of anesthesia (per min increase)	1.004 (0.998–1.010)	0.157	4.970 (1.431–17.264)	0.012
VDI (yes)^‡^	5.983 (1.355–26.414)	0.018	4.905 (1.079–22.307)	0.040
The ratio of postoperative NLR/preoperative NLR ≥ 1.86^§^	4.418 (1.685–11.582)	0.003	3.775 (1.398–10.192)	0.009

CI, Confidence interval; ASA, American Society of Anesthesiologists; NLR, Neutrophil-to-lymphocyte ratio.

^*^Education level was divided into 5 classes: illiterate (as reference), elementary school, middle school, high school, and college and above.

^†^A 30-score scale with higher score for better cognitive function.

^‡^Defined as serum 25(OH)-vitamin D < 50 nmol/L.

^§^Acute systemic inflammation was defined as the ratio of postoperative NLR/preoperative NLR ≥ 1.86 (the cutoff value for the risk of developing DNR in patients without VDI derived from Receiver operating characteristic analysis).

**Figure 2:**
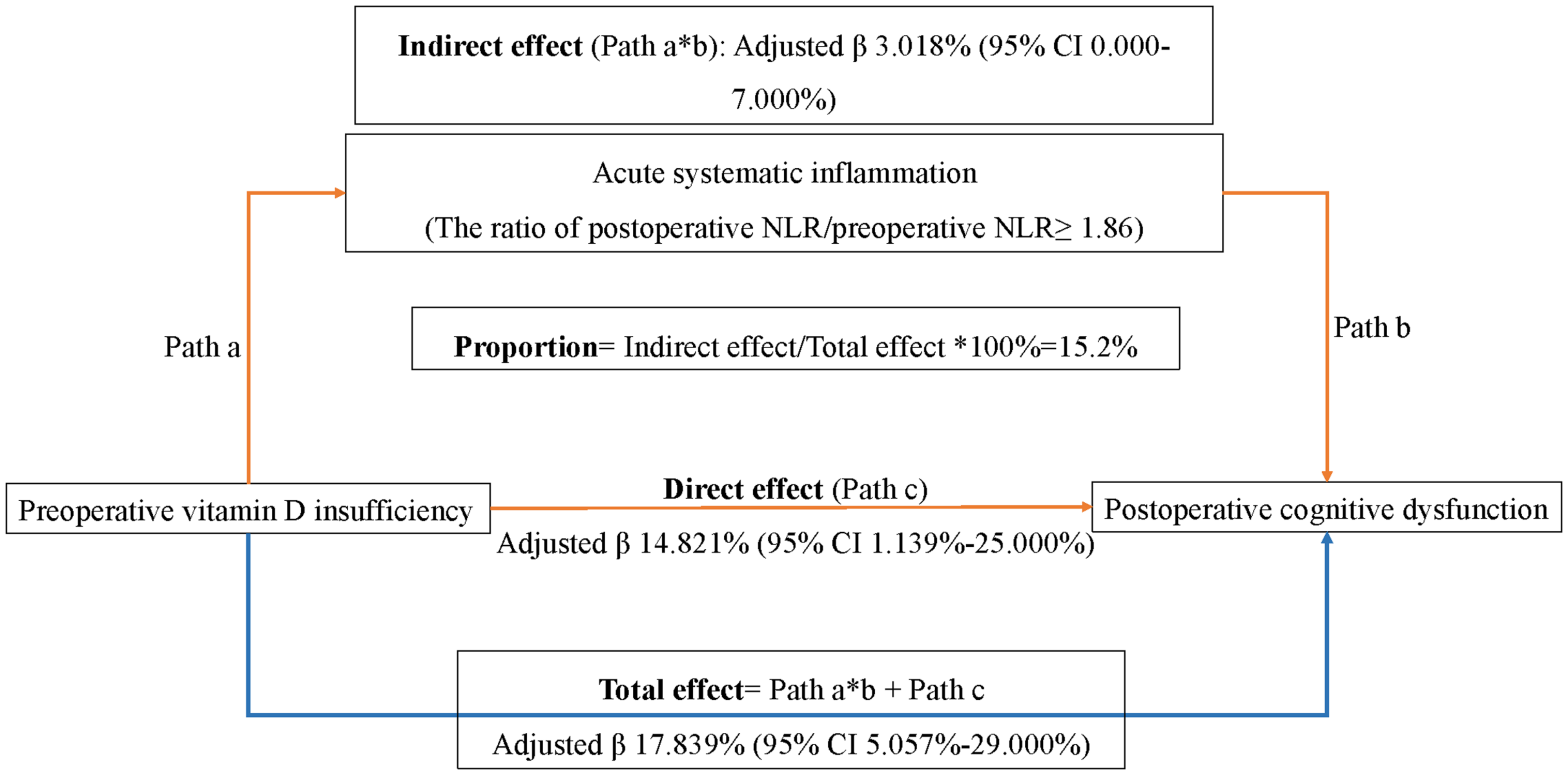
Mediation analysis. The association between VDI and DNR was significantly mediated by acute systemic inflammation (adjusted β 3.018%, 95% CI 0.000% to 7.000%, *P* = 0.045) and accounted for 15.2% of total effect. This result indicated that the incidence of DNR might be 3% higher in VDI patients if they suffered heavier systemic inflammation. Acute systemic inflammation was defined as the ratio of postoperative NLR/preoperative NLR≥ 1.86 (the cutoff value for the risk of developing DNR in patients without VDI derived from Receiver operating characteristic analysis).

After an adjustment of the confounders, mediation analysis showed that the total effect and direct effect of VDI on DNR was 17.839% (95% CI 5.057–29.000%, *p* = 0.016) and 14.821% (95% CI 1.139–25.000%, *p* = 0.038), respectively (Figure 2). The association between VDI and DNR was significantly mediated by acute systemic inflammation (adjusted β 3.018, 95% CI 0.000 to 7.000%, *p* = 0.045) and accounted for 15.2% of total effect. This result indicated that the incidence of DNR might be 3% higher in VDI patients if they suffer from heavier systemic inflammation.

## Discussion

The present study found that preoperative VDI was associated with an increased risk of DNR in elderly women who underwent major gynecological surgery. The ratio of postoperative NLR/preoperative NLR ≥ 1.86 was also associated with an increased risk of DNR, which led us to further discover that acute systemic inflammation may have served as a partial mediator in the possible impact of VDI on DNR. Meanwhile, VDI was not associated with POD.

The MoCA has been widely used to assess perioperative cognitive function. In comparison with mini mental-state examination, the MoCA is more sensitive to diagnose cognitive decline in elderly surgical patients (36). Several methods have been advocated to diagnose DNR using the MoCA score, such as the cut-off point (i.e., ≤26) and the 2 scores decline from baseline. Taking a battery of neuropsychological tests as reference, the method of 2 scores decline from baseline shows an optimal sensitivity (i.e., 82.6%) and specificity (i.e., 82.2%) (28). Thus, present study used this method to diagnose DNR. Amongst the enrolled patients, 7.7% (12/156) of them were illiterate and their MoCA scores were adjusted according to their education level (27).

The incidence of VDI was approximately 73.1% in this study, which was similar to previous results (9, 10). The present study focused on elderly women who were at a high risk of VDI because of postmenopausal hormone change and reduced outside activity (37). In this study, the proportion of low daily activity (<0.5 h per day) was significantly higher in patients with VDI. Another reason for this might be the insufficient intake of vitamin D. One nationwide study reported that only 10% of postmenopausal women received vitamin D supplementation (38).

Perioperative vitamin D supplementation may significantly increase the serum vitamin D concentration and inhibit the expression of inflammatory mediators, but there is lack of data to support its effect on DNR (14, 39). In critical care unit, patients treated with vitamin D3 (i.e., a single dose of 540,000 IU) at admission had better long-term global cognition and executive function (40). In surgical patients, perioperative supplementation of vitamin D may reduce the risk of postoperative pain and atrial fibrillation (41–43). While preoperative vitamin D screening may identify patients at potentially higher risk, current evidence is insufficient to definitively conclude impact of vitamin D supplementation on the development of DNR. Further research, ideally through randomized trials, is needed to establish causality and determine the efficacy and optimal strategy of perioperative vitamin D supplementation in surgical patients.

In this study, the ratio of postoperative NLR/preoperative NLR was used to reflect the severity of acute systemic inflammation changes, which were highly associated with cognitive function (44). In surgery patients, both preoperative NLR and postoperative NLR were independent risk factors of DNR (45, 46). However, these indicators could not reflect the acute change in systematic inflammation, especially the severity of change. In previous studies, the positive dose-response association between the level of NLR and the risk of DNR was established in surgery patients and stroke patients (45–47). Thus, we employed the ratio of postoperative NLR/preoperative NLR to reflect the severity of acute systemic inflammation variation.

Current findings highlight systemic inflammation as a key mediator linking VDI to DNR in elderly surgical patients. Recent evidence shows nutritional interventions attenuate inflammation-mediated outcomes (48), while handgrip strength interacts with nutritional status to affect recovery (49). This indicates VDI operates within a complex network of interrelated functional, nutritional, and inflammatory determinants influencing postoperative neurocognition. Integrating multimodal assessments—inflammatory biomarkers, nutritional status, and functional reserve—into preoperative geriatric evaluations may improve risk stratification and personalize perioperative management to mitigate DNR risk.

The present study found that acute systemic inflammatory responses may have served as a partial mediator in the correlation between VDI and DNR. The association between VDI and DNR has also been reported in elderly surgical patients undergoing major non-cardiac surgeries (13). In animal models, VDI may aggravate cognitive dysfunction caused by surgery and anesthesia via activating inflammatory response and enhancing cholinergic activity (50, 51). However, few clinical studies have focused on the association between VDI, acute systemic inflammatory, and DNR in surgical patients. In community-dwelling populations, it has been validated that VDI can increase the risk of dementia by activating neuroinflammatory responses (23, 52). On the other hand, acute systemic inflammation may enhance neuroinflammatory (24). Both our study and previous study found that VDI patients might suffer heavier inflammatory response than non-VDI patients (41). It is worthy noting that, the mediated effect size (indirect effect adjusted *β* = 3.018%) is modest in absolute value. However, even a small mediated proportion can be mechanistically informative. This finding suggests systemic inflammation plays a partial, yet statistically significant, role in explaining the link between VDI and DNR. This provides a potential biological pathway worthy of further investigation, even if the direct effect of VDI remains dominant.

The present study found that VDI was not associated with POD. This can be attributed to two reasons. Firstly, VDI was not severe enough to cause delirium. One meta-analysis showed that vitamin D deficiency (i.e., serum 25(OH)D < 25 nmol/L), but not insufficiency, was associated with increased risk of delirium (13). Secondly, the sample size of this study might be underpowered to test the association between VDI and delirium, because the incidence of delirium was significantly lower than DNR.

It is reported that VDI is associated with increased risks of postoperative organ injuries, such as infectious complications, acute kidney injury, and lung injury (53, 54). However, this was not observed in the present study. In line with previous studies, the length of postoperative in-hospital stay and the medical expenses were similar between the two groups (55, 56).

Our findings concerning preoperative VDI and DNR are specifically applicable to elderly women undergoing major gynecological surgery. Caution should be exercised when extrapolating these results to mixed-gender populations, given changes in estrogen and androgen levels during the aging process exert sex-specific variations in vitamin D metabolism (57), which may further alter the association strength between VDI and DNR. For other surgical types, the magnitude and nature of surgical trauma elicit inflammatory responses of varying degrees, which may attenuate the observed association.

The present study had several limitations. Firstly, the study was unable to characterize the severity and temporal dynamics of surgery-induced declines in vitamin D levels. This limitation arose from resource constraints and participant reluctance toward undergoing serial blood sampling. Consequently, potential selection bias may have been introduced, and the study was unable to draw definitive conclusions regarding the trajectory of perioperative vitamin D status—a phenomenon documented in previous research (41, 53, 58). Future studies should implement dedicated biomarker sampling protocols to better elucidate the impact of perioperative vitamin D fluctuations on clinical outcomes. Secondly, the sample size was insufficient to reliably assess the association between VDI and postoperative complications. This limitation compromises the statistical power and precision of the effect estimates, thereby increasing the likelihood of Type II errors—specifically, the failure to detect clinically relevant associations. Thirdly, vitamin D levels exhibit seasonal fluctuations primarily driven by variations in sunlight exposure. Clinical testing and interventions should be interpreted within their seasonal context. Given the study’s scope and sample size limitations, we did not adjust for seasonality in present research. Finally, we acknowledge that restricting cognitive assessment to a 5 day follow-up period limits the ability to draw conclusions regarding long-term neurological outcomes. These methodological constraints highlight the need for larger, prospective studies to confirm and expand upon these preliminary findings.

## Conclusion

This study established an association between preoperative VDI and an increased risk of DNR in elderly women who underwent major gynecological surgery. Acute systemic inflammatory responses may have served as a partial mediator in this correlation. These present findings indicate preoperative VDI screening may aid risk stratification for DNR. Given inflammation’s possible mediating role, targeted perioperative interventions—such as optimized analgesia, early mobilization, or anti-inflammatory agents—may reduce DNR incidence in VDI patients. Combining preoperative screening with inflammatory modulation may improve both short-term recovery and long-term cognitive outcomes in this high-risk population.

## Data Availability

The original contributions presented in the study are included in the article/Supplementary material, further inquiries can be directed to the corresponding authors.
