# Supplementary material for: Preoperative vitamin D insufficiency increases the risk of delayed neurocognitive recovery via acute systemic inflammation in elderly women undergoing gynecological surgery
Source: Front Med (Lausanne). 2026 Feb 18;13:1626647. doi: 10.3389/fmed.2026.1626647 (PMC12956702; doi:10.3389/fmed.2026.1626647)
Supplement: Supplementary file 1 [file Table_1.docx]

Supplementary Material

# Supplemental Table 1 Universal analyses of all potential factors in association with delayed neurocognitive recovery

| **Variable** | **Univariate analysis** | |
| --- | --- | --- |
|  | **Odds Ratio (95% CI)** | **P value** |
| **Baseline variables** |  |  |
| Age (per year increase) | 0.994（0.912-1.083） | 0.885 |
| Body mass index (per kg/m^2^ increase) | 1.026（0.909-1.157） | 0.680 |
| Education level (per level increase) ^*^ | 0.715（0.510-1.003） | **0.052** |
| ASA physical status classification (%) |  |  |
| II | Reference |  |
| III | 0.861（0.379-1.957） | 0.721 |
| Married (yes) ^†^ | 0.603（0.244-1.490） | 0.273 |
| Stroke (yes) | 0.313（0.039-2.496） | 0.273 |
| Hypertension (yes) | 1.597（0.688-3.705） | 0.276 |
| Arrhythmia (yes) ^‡^ | 0.267（0.034-2.105） | 0.210 |
| Diabetes (yes) | 1.180（0.475-2.929） | 0.722 |
| Thyroid disease (yes) | 2.815（0.633-12.525） | 0.174 |
| Hyperlipidemia (yes) | 1.521（0.625-3.700） | 0.355 |
| Musculoskeletal disorders (yes) ^¶^ | 0.969（0.302-3.113） | 0.958 |
| Barthel index (per score increase) ^§^ | 1.026（0.924-1.139） | 0.631 |
| Duration of daily activity (%) |  |  |
| < 0.5 hour | reference |  |
| 0.5-2 hours | 1.183（0.504-2.775） | 0.699 |
| ≥ 2 hours | 1.848（0.435-7.847） | 0.405 |
| Daily calcium supplementation within recent 3 months (yes) | 0.850（0.295-2.449） | 0.763 |
| Montreal cognitive assessment (per score increase) ^ΙΙ^ | 0.862（0.777-0.957） | **0.005** |
| Vitamin D insufficiency (yes) ^#^ | 0.270（0.077-0.947） | **0.041** |
| Calcium (per mmol/l increase) | 2.566（0.026-256.383） | 0.688 |
| Magnesium (per mmol/l increase) | 0.296（0.001-81.463） | 0.671 |
| Phosphorus (per mmol/l increase) | 0.972（0.804-1.174） | 0.767 |
| C-reactive protein (per mg/l increase) | 1.005（0.963-1.050） | 0.807 |
| Hemoglobin (per g/l increase) | 1.023（0.987-1.060） | 0.209 |
| **Perioperative variables** |  |  |
| Anesthesia type (general anesthesia [GA] vs. GA+ paravertebral block) | 0.468（0.057-3.849） | 0.480 |
| Anesthesia time (per minute increase) | 1.005（0.999-1.011） | 0.132 |
| Surgery time (per minute increase) | 1.004（0.997-1.011） | 0.298 |
| Sufentanil (per μg increase) | 1.004（0.994-1.014） | 0.455 |
| Remifentanil (per μg increase) | 1.001（1.000-1.002） | 0.140 |
| Use of midazolam (yes) | 1.247（0.791-1.965） | 0.342 |
| Use of sevoflurane (yes) | 0.529（0.234-1.198） | 0.127 |
| Total fluid input (per ml increase) | 1.000（1.000-1.001） | 0.525 |
| Estimated blood loss (per ml increase) | 1.001（0.997-1.004） | 0.742 |
| Intraoperative hypotension (yes) | 1.344（0.550-3.283） | 0.517 |
| Intraoperative hypoxemia (yes) | 0.000（0.000-） | 0.999 |
| Intraoperative hypertension (yes) | 1.519（0.578-3.993） | 0.397 |
| Use of patient-controlled intravenous analgesia (yes) | 1.409（0.298-6.667） | 0.666 |
| Use of non-steroid anti-inflammatory drugs (yes) | 0.509（0.190-1.362） | 0.179 |
| Pain intensity at movement (per score increase) ^**^ |  |  |
| Postoperative 1^st^ day | 0.920（0.769-1.101） | 0.363 |
| Postoperative 2^ed^ day | 0.988（0.810-1.207） | 0.908 |
| Postoperative 3^th^ day | 0.968（0.768-1.219） | 0.781 |
| Sleep quality (per score increase) ^††^ |  |  |
| Postoperative 1^st^ night | 1.110（0.944-1.306） | 0.207 |
| Postoperative 2^ed^ night | 0.889（0.714-1.109） | 0.297 |
| Postoperative 3^th^ night | 0.859（0.685-1.077） | 0.187 |
| Preoperative NLR ^‡‡^ | 0.952（0.687-1.320） | 0.770 |
| Postoperative NLR ^‡‡^ | 1.006（0.957-1.057） | 0.827 |
| The ratio of postoperative NLR /preoperative NLR≥ 1.86 ^¶¶^ | 4.418 (1.685-11.582) | **0.003** |

Data were presented as odds ratio (95% confidence interval). ASA= American Society of Anesthesiologists, NLR= neutrophil/lymphocyte ratio.

^*^ Education level was divided into five classes: illiterate, elementary school, middle school, high school, and college and above.

^†^ Married participants versus those who was divorced or widowed.

^‡^ Including premature ventricular complexes, premature atrial contraction, and I ^◦^ atrioventricular block.

^¶^ Including spinal spondylolysis and joint osteoarthrosis.

^§^ A 100-score scale with higher score for better daily activity.

^ΙΙ^ A 30-score scale with higher score for better cognitive function.

^#^ Serum 25-hydroxyvitamin D < 50 nmol/l.

^**^ Evaluated by numeric rating score (11-score, 0 for no pain and 10 for worst pain).

^††^ Evaluated by numeric rating score (11-score, 0 for worst sleep and 10 for good sleep).

^‡‡^ The neutrophil/lymphocyte ratio (NLR) is a reproducible marker of systemic inflammatory responses.

^¶¶^ Acute systemic inflammation was defined as the ratio of postoperative NLR/preoperative NLR≥ 1.86 (the cutoff value for the risk of developing delayed neurocognitive recovery in patients without VDI derived from Receiver operating characteristic analysis).
